# Discharge Against Medical Advice From the Emergency Department: Results From a Private Hospital in Beirut

**DOI:** 10.7759/cureus.79800

**Published:** 2025-02-27

**Authors:** Danielle Abou Khater, Joelle Kalaji, Alain Tanios, Charbel Ghosn, Robert Fakhoury, Mariana Helou

**Affiliations:** 1 Department of Emergency Medicine, Lebanese American University Medical Center, Beirut, LBN; 2 Department of Emergency Medicine, Lebanese American University School of Medicine, Beirut, LBN; 3 Department of Cardiology, Lebanese American University School of Medicine, Beirut, LBN

**Keywords:** against medical advice, discharge, economic crisis, emergency, emergency department

## Abstract

Introduction: Discharge against medical advice (AMA) is a common problem worldwide. These patients experience higher mortality rates in the following month and higher Emergency Department (ED) revisit rates. This study examines the characteristics, reasons, and clinical outcomes of the patients leaving the ED AMA.

Methods: This is a cross-sectional study conducted at the ED of the Lebanese American University Medical Center from 2019 to 2022.

Results: Over the four years, 42,672 patients have presented to the ED. Among them, 2,767 have left AMA (6.4%). The numbers varied among the years, from 477 (3.6%) in 2019, going up to 751 (7%) in 2020, then to 907 (10%) in 2021, and 632 (5.8%) in 2022. Many reasons were found. The most common reason for leaving AMA was the financial coverage, which accounted for 1442 cases (52%). Other common causes were the COVID-19 isolation cost (started in 2020) with 677 cases (24.5%), cold cases referred to clinics (301 cases; 10.9%), and the long waiting time for a bed being available (284 cases; 10.3%). Other causes were only 63 (2.3%).

Conclusion: Discharge AMA varies from one ED to another but is mainly linked to the economic situation in the country, the financial coverage of the population, and the system within the hospital. Interventions at a larger scale shall be conducted to reduce the rate of its occurrence.

## Introduction

One of the biggest challenges facing the healthcare system is discharge against medical advice (AMA) from Emergency Departments (EDs), with a global prevalence rate between 0.07 and 20% [1,2]. It occurs when patients are discharged from the hospital against the advice of their healthcare provider to remain for further treatment. Patients who leave AMA face a higher risk of adverse health outcomes, often facing more costly and intensive healthcare needs down the line due to unresolved medical conditions or subsequent complications [3-5]. AMA discharges are associated with an increased relative risk of mortality as high as 10% at one month and a higher 30-day readmission rate compared to the readmission rate following standard discharge [4-9]. It is critical to understand the reasons behind patients’ refusal of admission and discharges AMA to improve patient health outcomes and reduce the strain on medical services. Retrospective studies showed that higher AMA discharges were linked to younger age, male gender, history of substance use, psychiatric illness, lack of medical health insurance, low socioeconomic status, and lack of education [4,6-8,10-20].

Various reasons were found for patients leaving the ED, including family commitment, having children at home, financial problems, negative interaction with the ED staff, lack of medical improvement, disagreement with the procedure plan, and long ED waiting time [2,5,21-26].

Lebanon has faced mounting challenges since the country’s economic crisis began in 2019. This crisis has led to hyperinflation, mass unemployment, and a drastic reduction in the availability of essential resources, placing an unusual burden on the healthcare system. The already fragile country also had to deal with the aftermath of the COVID-19 pandemic and the devastating Beirut Port explosion in 2020 [27,28]. These compounding crises have severely impacted the healthcare sector, reducing access to essential services and straining hospital resources [27,28]. Therefore, many patients are unable to afford the dramatically rising medical expenses, prompting them to delay or avoid seeking treatment altogether. In the context of emergency care, this financial burden may result in patients leaving without being seen by a physician or AMA of admission for treatment due to their inability to afford the necessary medical services, lack of insurance coverage, or fear of accumulating medical debt. Not only is patient safety compromised after such discharges, but there is also additional stress placed on emergency care providers who are challenged with cases where treatment must be left incomplete or prematurely stopped due to financial constraints [3,4].

Studies done in low-income and middle-income countries also suffering from economic hardships have shown a comparable trend, where financial concerns often drive patients to refuse care, leaving hospitals without being seen or AMA after being informed about their need for admission [23,29,30]. However, there is limited research on the specific role of economic crises in influencing AMA discharges, particularly in Lebanon, where the specific impact of the ongoing crisis remains underexplored.

The aim of this study is to identify the primary reasons behind AMA discharges in Lebanese EDs, with a particular focus on the impact of the ongoing economic crisis on these decisions. By addressing these reasons, this study can offer insights to policymakers, hospital administrators, and healthcare providers on how to mitigate preventable AMA discharge and improve patient care, therefore reducing the subsequent complications following incomplete treatment resulting from AMA discharges.

## Materials and methods

The Lebanese American University Medical Center (LAUMC) is a private teaching university hospital in Beirut with an estimated 1500 ED visits per month. This cross-sectional study was conducted at the ED of LAUMC. All patients presenting to the ED are triaged by the triage nurse, an admitting file is opened, and the patient is admitted inside the ED to be examined and assessed by the ED resident/ED physician. Any patient who refuses the medical care or medical advice for admission or procedure and wants to leave before management or treatment is completed will have to sign the against medical advice form.

The study is a cross-sectional study. All medical records of 42,672 patients who were admitted to the ED from January 2019 to December 2022 were reviewed. All patients who either left without being seen by a physician or discharged themselves AMA after signing the preprinted against medical advice form were included in the study. We excluded patients aged less than 18 years. The data was retrieved from the patients’ charts uploaded in the hospital database system and the ED database. The data recorded included patients’ characteristics and reasons for leaving AMA. All data collected during the study remained anonymous, ensuring participants could not be identified. Furthermore, only authorized members of the research team had access to the data.

Statistical analyses were performed using IBM SPSS Statistics for Windows, Version 27 (Released 2020; IBM Corp., Armonk, New York, United States). Descriptive statistics were calculated for the total study sample, with numbers and percentages for all the variables. This study was approved by the Institutional Review Board of the Lebanese American University (IRB # LAUMCRH.MH2.21/Nov/2024).

## Results

Over the four years, 42,672 patients have presented to the ED. Among them, 2,767 have left AMA (6.4%). These patients had an average age of 52 years old.

**Figure 1:**
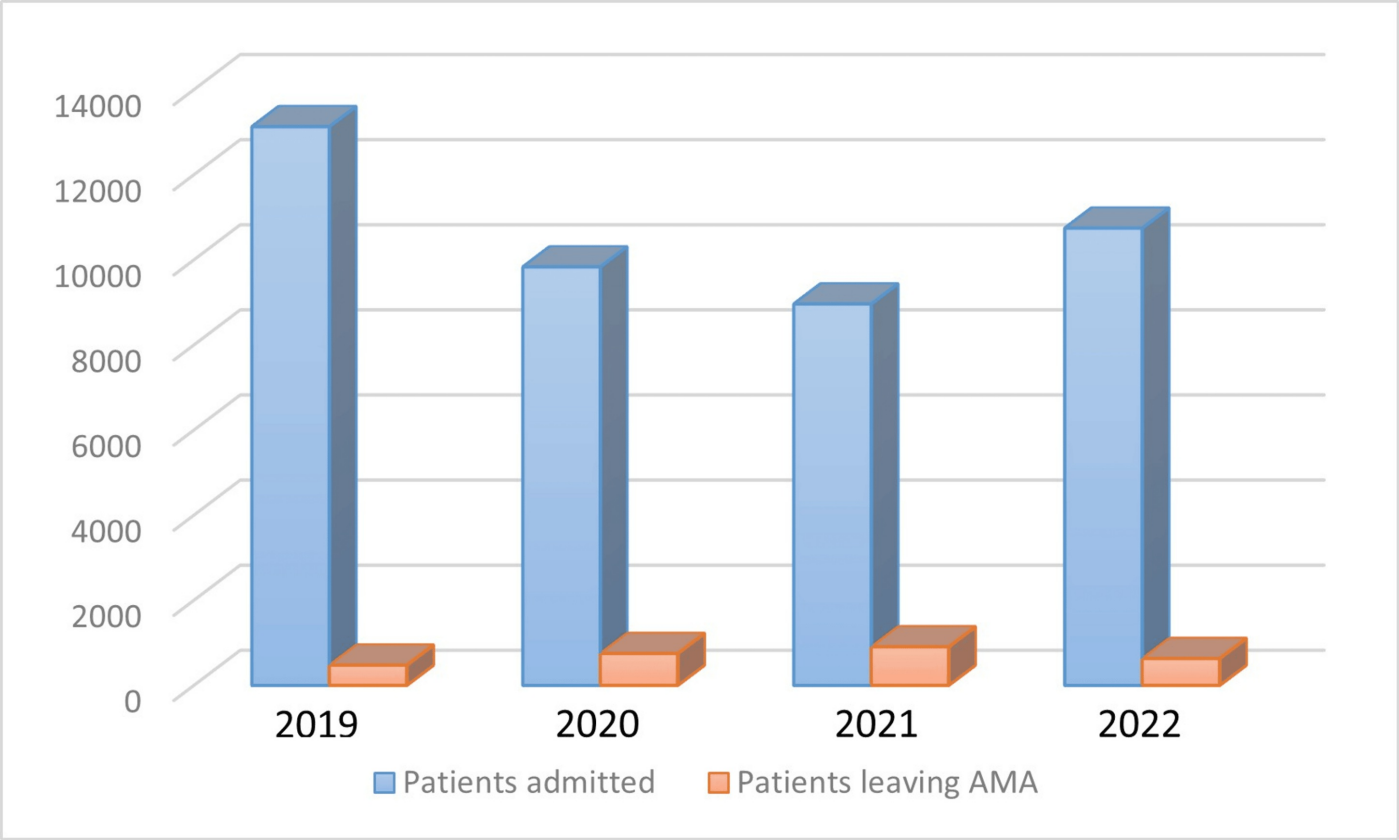
Number of patients leaving against medical advice AMA: Against medical advice

The numbers varied among the years, from 477 (3.6%) in 2019, going up to 751 (7%) in 2020, then to 907 (10%) in 2021, and 632 (5.8%) in 2022. As illustrated in Figure 1, the total number of patients leaving AMA is compared to the total number of patients admitted to the ED. In 2019, 477 patients left AMA from a total of 13130 patients admitted to the ED (3.6%); in 2020, 751 left AMA from 9834 ED admissions; in 2021, 907 left AMA from 8963 ED admissions, and in 2022, 632 left AMA from 10745 ED admissions.

The most common reason for leaving AMA was the financial coverage, which accounted for 1442 cases (52%). Other common causes were the COVID-19 isolation cost (started in 2020) with 677 cases (24.5%), cold cases referred to clinics (301 cases; 10.9%), and the long waiting time for a bed being available (284 cases; 10.3%). Other causes were only 63 (2.3%).

## Discussion

Over the four years, on average, 6.4% of the patients have left AMA with percentages varying across the years. This percentage was similar to that in some other institutions, such as 5.96 % in Kathmandu [23]. Percentages can go lower, as in Bahrain 2.5 % [5], Ghana 2.5 % [31], and the US 1.6% [20].

A similar study conducted in another hospital in Lebanon in 2012 found 1213 patients leaving AMA in an ED with 49000 ED visits per year [2]. The study does not report the exact total number of patients presented this year at the hospital. However, the percentage can be calculated to be around 25%, which is higher than what is found in other studies. The main increase was seen in 2021, with a percentage of 10%. This increase in 2020 and 2021 came along with the economic collapse that hit the country at the end of 2019 [32,33]. The issue of discharges AMA in Lebanon's ED is closely tied to the country's economic crisis, making it a critical challenge for both healthcare providers and policymakers. The economic downturn has significantly impacted the healthcare system, with hyperinflation and unemployment exacerbating patients' inability to afford necessary treatments. This financial strain has made hospital visits financially prohibitive for many, pushing patients to decline or prematurely discontinue care even when medical professionals strongly recommend further treatment. As seen in other low- and middle-income countries facing similar economic difficulties, financial hardship is often a major driver behind patients’ decisions to leave AMA. In Lebanon, the high cost of healthcare, lack of insurance coverage, and fear of insurmountable medical debt are prevalent factors influencing patients to leave AMA. These discharges are not only a risk to patient safety, potentially leading to worsening health conditions and future emergencies, but they also place an additional burden on healthcare providers who face ethical dilemmas and resource constraints as they manage cases that may go untreated.

In our study, the most common cause for leaving AMA was the financial coverage, similarly seen in many other studies [2,5,21-26].

Addressing AMA discharges in Lebanon’s context requires systemic interventions that go beyond the hospital setting, involving government efforts to stabilize the healthcare economy, increase insurance coverage, and implement policies that make healthcare accessible and affordable for all. Additionally, hospitals may benefit from implementing financial support programs or deferred payment options to reduce the likelihood of AMA discharges. A better understanding of the economic and psychosocial factors contributing to AMA discharges can inform such policies, ultimately improving patient outcomes and alleviating the strain on Lebanon’s healthcare system during these challenging times.

Limitations

The study was conducted in one single private hospital, which limits the results to a single center. However, the data was retrieved over four years to recruit as many patients as possible. Also, our hospital is a major hospital in the capital city Beirut, with a large catchment area. This makes our results generalizable to hospitals with similar payment modalities.

Another limitation of this study is that patients were not followed up after their discharge, so no data could be available for morbidity or mortality. We also add the inability to establish causation due to the cross-sectional nature of the study. Our results are mainly descriptive in nature, and these results can help address the issues that needed intervention. A prospective study would be beneficial to conduct later on to assess these patients and confirm causality.

## Conclusions

Rates of discharge AMA differ significantly from one ED to another depending on multiple factors. Key contributors include the socioeconomic and political situation in the country, the financial coverage available for the population, and hospital-specific policies. Addressing this issue requires interventions from the hospital administrators and at a larger scale to reduce the frequency of occurrence of AMA discharges, therefore limiting its consequences on the patients and the healthcare system and consequently promoting better health outcomes.
